# Hidden in plain sight: why diagnosing asymptomatic congenital cytomegalovirus (cCMV) changes everything

**DOI:** 10.1016/j.bjorl.2025.101728

**Published:** 2025-12-02

**Authors:** Vagner Antonio Rodrigues Silva, Nicolau Moreira Abrahão, Arthur Menino Castilho

**Affiliations:** Universidade de Campinas (UNICAMP), Faculdade de Ciências Médicas (FCM), Departamento de Otorrinolaringologia e Cirurgia de Cabeça e Pescoço, Campinas, SP, Brazil

## Introduction

Congenital cytomegalovirus (cCMV) infection represents the most common congenital infection worldwide, with incidence rates ranging from 4.8 to 14.2 per 1000 live births. As one of the leading preventable causes of neurological disability and childhood hearing loss, cCMV is responsible for 6%–30% of pediatric hearing loss cases.

The clinical challenge lies in the fact that approximately 85%–90% of infected children are asymptomatic at birth yet face significant risks of late-onset and progressive sequelae. These asymptomatic patients may present with sensorineural hearing loss from birth that progresses over time or develop it later during the first years of life, with an incidence of 22% and losses ranging from mild to profound.^1^^,^^2^

Since hearing loss typically begins late and manifests after three months of age, asymptomatic newborns are not detected through universal newborn hearing screening (UNHS), which should be performed within the first month of life. Approximately 60% of cases present bilateral hearing loss, while 40% are unilateral⁷. Unilateral loss, often underestimated, causes significant difficulties in sound localization, comprehension in noisy environments, and academic development.^2^^,^^3^

The hearing loss spectrum ranges from mild to profound, with the potential for fluctuating hearing thresholds. Some patients may experience episodes of sudden hearing loss. Cohort studies show an increased prevalence of autism characteristics in children with cCMV, especially those with associated hearing loss. The prevalence of autism spectrum disorder (ASD) may be 2–3 times higher in children with cCMV compared to the general population.^4^

## Diagnostic methods: critical performance comparison

The selection of appropriate diagnostic methods is fundamental for establishing effective screening programs (Table 1).^1^^,^^5^^,^^6^

**Table 1:** Comparative table of diagnostic methods.

Method	Sensitivity (%)	Specificity (%)	PPV (%)	NPV (%)	Main limitations
Urine (PCR)	93%−100%	100%	100%	99.8%–99.9%	• Difficult collection
					• Requires sterile technique
					• Inadequate volume in newborns
Saliva (PCR)	85%–97%	99%–100%	86.7%–100%	99.9%–100%	• Risk of contamination
					• False-positives
					• Confirmation required
DBS	73%–85.7%	100%	97.7%–100%	75%–99.9%	• Low sensitivity
					• Variable viremia
					• Diagnostic window
Cord blood	24%–70%	90%–100%	Variable	Low	• Low sensitivity
					• Limited volume
					• Restricted availability

PPV, Positive Predictive Value; NPV, Negative Predictive Value; PCR, Polymerase Chain Reaction; DBS, Dried Blood Spots.

**Urine PCR - Gold Standard** - The gold standard for diagnostic confirmation, offering the best combination of sensitivity (93%−100%) and specificity (100%). The critical diagnostic window of 3 weeks makes early collection essential. Main limitations include difficult collection, the need for sterile technique, and inadequate volume in newborns.

**Saliva PCR - Preferred Screening Method** - Ideal method for universal screening due to ease of collection. Presents higher viral loads than urine (median 2 × 10⁶ vs 8 × 10⁵ IU/mL), with sensitivity of 85%−97% and specificity of 99%−100%. Requires confirmation by urine due to the risk of false positives from contamination.

**Dried Blood Spots (DBS) -** Useful for retrospective diagnosis, especially in children who failed neonatal hearing screening. Performance varies significantly with viremia, being more effective in symptomatic cases. Sensitivity ranges from 73% to 85.7%, with 100% specificity, but is limited by variable viremia and the diagnostic window.

**Umbilical Cord Blood** - Demonstrates low sensitivity (24%−70%), inadequate for routine clinical use. Reserved for specific research situations due to limited volume and restricted availability.

## Recommended diagnostic algorithm

Universal screening using saliva PCR demonstrates 90% sensitivity and 100% specificity. Alternatively, targeted screening of newborns who fail neonatal hearing screening represents a more feasible and cost-effective strategy.^1^^,^^5^

### Screening cCMV algorithm (Fig. 1)

Educational programs about hygiene measures during pregnancy, especially for women in contact with young children, constitute an essential primary prevention strategy.

**Fig. 1:**
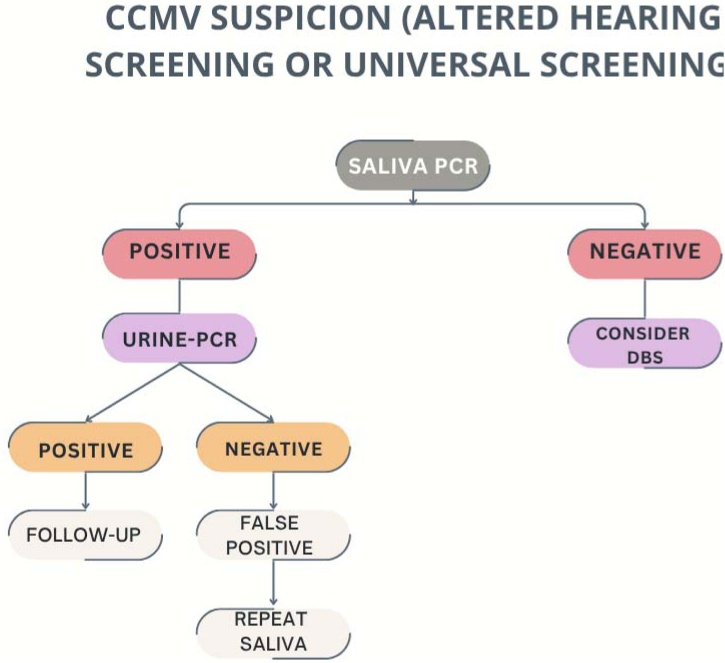
Screening cCMV Algorithm. cCMV, Congenital Cytomegalovirus; PCR, Polymerase Chain Reaction; DBS, Dried Blood Spots.

### Suggested follow-up protocol

Confirmed diagnosis of asymptomatic cCMV should trigger a specialized follow-up protocol that differs radically from routine otolaryngological monitoring. Since the risk of hearing loss is higher in these patients, screening examination with otoacoustic emissions and automated auditory brainstem response (AABR) in the neonate is important.

### Serial audiological follow-up

• **0–6 months:** AABR every 3 months for early detection of changes.
• **6–24 months:** AABR every 6 months if previous exams are normal.
• **2–5 years:** Annual AABR, even with previously normal hearing. From 4 years of age, audiometry and impedance testing may be possible.
• **School age:** Annual audiometry.

Any alteration in hearing thresholds should be investigated immediately, including magnetic resonance imaging (MRI) of the inner ear.

### Auditory rehabilitation

• Early amplification in cases of hearing loss, even mild unilateral.
• Consider cochlear implantation for severe/profound losses, especially in unilateral hearing losses, due to the higher risk of contralateral ear hearing loss in the future.
• Auditory rehabilitation therapy with a specialized speech-language audiologist.

The intensified follow-up requires service restructuring, including priority scheduling for serial audiological evaluations, integration between audiology, neurology, and developmental services, and creation of specific institutional protocols. Cost-effectiveness is favorable when compared to the costs of late rehabilitation and special education. Early detection of hearing loss allows intervention during the critical period of language development.

## Management considerations and limitations

It is essential to acknowledge the limitations in cCMV management. Not all children will benefit from antiviral treatment, and the criteria for therapeutic indication are still evolving. Diagnosis may generate family anxiety, requiring adequate counseling and psychological support.

The paradigm of follow-up transformed by asymptomatic cCMV diagnosis, with its intensified audiological evaluations, directed neuroimaging, and specialized early interventions, offers a unique opportunity to positively alter the neurological and auditory developmental trajectory of these children.

## Conclusion

The diagnosis of cCMV in asymptomatic children is essential and critical because it can determine a child's auditory and neurological development. The apparent initial clinical normalcy should never be interpreted as the absence of future risk. Each child with undiagnosed asymptomatic cCMV represents a lost opportunity to implement a follow-up protocol that can prevent or minimize permanent auditory and neurological deficits.

Asymptomatic cCMV is not a benign condition requiring only observation, but rather a condition demanding proactive, specialized follow-up, and targeted early intervention. The cost of inaction is measured not only in resources but mainly in the lost auditory, communicative, and cognitive potential of children who could have had significantly better outcomes with early diagnosis and adequate specialized follow-up.

The emerging evidence of a possible association with autism spectrum disorder adds a new dimension to follow-up complexity, requiring targeted screening and specialized multidisciplinary evaluation. This reality reinforces the need for reference centers with specific experience in cCMV.

Future research should focus on developing more effective therapies, identifying prognostic biomarkers, validating cost-effective screening strategies, and clarifying the possible association with autism spectrum disorder.

## ORCID ID

Vagner Antonio Rodrigues Silva: 0000-0002-7335-4489

Nicolau Moreira Abrahão: 0000-0002-0215-0948

Arthur Menino Castilho: 0000-0002-9024-8004

## Financial disclosure

None.

## Declaration of competing interest

The authors declare no conflicts of interest.
